# The fortification method relying on assumed human milk composition overestimates the actual energy and macronutrient intakes in very preterm infants

**DOI:** 10.1186/s40748-018-0090-4

**Published:** 2018-09-17

**Authors:** Israel Macedo, Luis Pereira-da-Silva, Manuela Cardoso

**Affiliations:** 1 NICU, Maternidade Dr Alfredo da Costa, Centro Hospitalar de Lisboa Central, Rua Viriato, n° 1, 1050-010 Lisbon, Portugal; 2 Medicine of Woman, Childhood and Adolescence, NOVA Medical School, Universidade NOVA de Lisboa, Lisbon, Portugal; 3 NICU, Hospital Dona Estefânia, Centro Hospitalar de Lisboa Central, Lisbon, Portugal; 4 Dietetics Unit, Maternidade Dr Alfredo da Costa, Centro Hospitalar de Lisboa Central, Lisbon, Portugal

**Keywords:** Human milk composition, Human milk fortification, Nutrient intake, Target fortification, Very preterm infants

## Abstract

**Background:**

To achieve recommended nutrient intakes in preterm infants, the target fortification method of human milk (HM) was proposed as an alternative to standard fortification method. We aimed to compare assumed energy and macronutrient intakes based on standard fortified HM with actual intakes relying on measured composition of human milk (HM), in a cohort of HM-fed very preterm infants.

**Methods:**

This study is a secondary retrospective analysis, in which assumed energy and macronutrient contents of daily pools of own mother’s milk (OMM) from 33 mothers and donated HM (DHM) delivered to infants were compared with the measured values using a mid-infrared HM analyzer. A fortification method consisting of modular protein and/or fat supplements added to standard fortified HM was used to provide the minimum recommended daily intakes of energy 110 Kcal/kg and protein up to 4.0 g/kg. Assumed nutrient intakes were compared with actual nutrient intakes from full enteral feeding to 35 weeks plus 6 days postmenstrual age, using the Wilcoxon matched-pairs signed ranks test.

**Results:**

The composition of 1181 samples of daily pools of HM were measured. For 90.2% of study days, infants were exclusively fed OMM and in remaining days fed OMM *plus* DHM. Comparing with reported preterm OMM composition, measured protein concentration was significantly lower, and energy and other macronutrient concentrations were lower only from the second to third postnatal week. Using fortified HM, the actual median daily intakes of energy, protein, and fat were significantly lower (113.3 vs. 120.7 Kcal/kg, 4.45 vs. 4.73 g/kg, and 4.96 vs. 5.35 g/kg, respectively) and the actual protein-to-energy ratio (PER) significantly higher than what was assumed (4.2 vs. 4.0), without differences in carbohydrate intake.

**Conclusions:**

When fortifying the HM, we used conservative target intakes trying not to exceed the osmolarity recommended for infant feeds. Actual energy, protein and fat intakes in OMM were significantly lower than assumed. This resulted in inadequate intake using our fortification method, that did not compensate the suboptimal measured energy and macronutrient contents of OMM delivered. Further studies comparing assumed with the gold standard target fortification are needed to determine safe upper limits of assumed fortification.

## Background

The American Academy of Pediatrics [1] and the European Society for Paediatric Gastroenterology, Hepatology, and Nutrition [2] recommend human milk (HM) as the first choice for feeding very preterm infants, provided it is fortified with nutrients necessary to meet requirements [2]. To provide adequate care for this infant population, strategies to prevent severe in-hospital nutrient deficits encompass the multicomponent fortification of HM [3]. However, the widely used standard fortification method, in which a fixed dose of nutrients is added independently of HM composition, rarely meets the recommended intake of protein for preterm infants [2], with inherent risks of growth faltering and neurocognitive impairment [3–6]. As an alternative, the targeted fortification method was proposed to tailor the individual infant’s needs, based on previous analyses of HM energy and macronutrients [7]. However, this method is time consuming and labor intensive, and analyzers are commonly unavailable [8, 9]. Other fortification methods of HM have been described, including the addition of modular protein and fat to fortified HM [10–13].

Some authors have compared fortification methods based on assumed HM composition with methods relying on measured HM composition [8, 11, 13, 14]. In these studies, differences between assumed and measured HM composition [8], assumed and actual intakes provided by fortified HM [8, 13], and growth [8, 11, 14] were assessed. Results from similar strategies have not been consistent, probably due to differences in methods of fortification and different characteristics of studied infants.

In our unit, logistical constraints related with reduction of health personnel precluded targeted fortification during a certain period of time, but samples of HM delivered to infants were stored frozen for later composition analysis. An alternative method of fortification was used, based on the assumed variable HM composition [3, 15]. Specifically, modular protein and fat supplements were added to standard fortified HM [11] to achieve recommended intakes [2].

In this study, we aimed to compare retrospectively the assumed energy and macronutrient intakes with the correspondent actual values relying on measured HM composition, in a cohort of HM-fed very preterm infants.

## Methods

This single-center study is a secondary retrospective analysis that used data from a birth cohort study aimed to determine the associations between macronutrient intake, body composition, and neurodevelopmental outcome in exclusively or predominantly HM-fed very preterm infants [7, 15]. The cohort study was performed in the neonatal intensive care unit (NICU) of Maternidade Dr. Alfredo da Costa, Centro Hospitalar de Lisboa Central, Lisbon, Portugal. The study was registered with the International Standard Randomized Controlled Trial Number (ISRCTN) registry (ID: 27916681) and approved by the Hospital Ethics Committee (ID 116/2012). Parents or guardians of all infants gave their informed consent before inclusion in the study, and the study was conducted in accordance with the tenets of the Declaration of Helsinki.

The characteristics of the 33 infants included are described elsewhere [6]. In brief, they were born at a mean (standard deviation [SD]) gestational age of 30 (1.8) weeks, with a median (interquartile range [IQR]) weight of 1175 (1010–1408) g. Infants were exclusively or predominantly HM-fed (formula-feeding < 12.5% enteral volume intake). Human milk fortification was initiated when enteral daily intake reached 100 mL/kg at a median (IQR) of 12 (11–14) postnatal days; full enteral feeding was achieved approximately at the same age, at a median (IQR) of 11 (8–16) postnatal days.

Mothers of studied infants were advised to sequentially collect milk every 3 h, either in the hospital or at home, and to record date and hour of each collection. The own mother’s milk (OMM) was stored frozen at − 25 °C in the maternity milk bank. For each infant, a daily pool of sequentially collected OMM batches, roughly representing the composition of OMM collected within a day, was thawed at 37 °C and mechanically homogenized to deliver to infants. A 3-mL sample from this pool was collected and again frozen for later analysis. Donated human milk (DHM) from mothers of term infants was frozen at home, transported frozen to the maternity milk bank, and stored at − 25 °C in the maternity milk bank. After negative screening for transmissible infectious diseases according to National Institute for Health and Care Excellence (NICE) guidelines [16], milk of each donor was thawed, pooled for microbiological control and macronutrient analysis, pasteurized using the Holder method [17], and frozen again. When DHM was necessary to complete the daily amount of prescribed HM, a pool of DHM was thawed using the aforementioned method.

A mid-infrared HM analyzer (Miris AB, Uppsala, Sweden) was used to measure the content of total protein, fat, carbohydrates, and energy of OMM. This is reported to be an accurate method to measure HM composition, validated and calibrated against chemical analysis for nitrogen and fat [13, 18]. Before analysis, samples of native OMM and DHM were thawed by warming to 40 °C and ultrasonically homogenized. As almost all infants were breastfed (unknown volume intake and composition) by 35 weeks plus 6 days postmenstrual age (PMA), the OMM analysis was suspended at this age.

Following the nutrition protocol used in our unit, we assumed in preterm OMM averages of 1.1 g/100 mL of protein in the first 3 postnatal weeks and 0.8 g/ 100 thereafter; in DHM, we assumed 0.8 g/ 100 mL of protein, and 67 kcal/ 100 mL of energy in both OMM and DHM. A fortification method consisting of modular protein and fat supplements added to standard fortified HM was used to provide the minimum recommended daily intakes according to body weight, as follows: energy 110 kcal/kg; protein (g/kg) 4.0 if < 1000 g, 3.7 if < 1200 g, 3.6 if < 1800 g, and 3.4 if > 1800 g; and protein-to-energy ratio (PER) of 3.6 if < 1000 g, 3.2 if < 1800 g, and 2.6 if > 1800 g [2, 3, 19]. The composition of the HM fortifier (Aptamil FMS®; Milupa/Danone GmbH, Friedrichsdorf, Germany), modular protein (Aptamil Protein Supplement powder®; Milupa/Danone GmbH, Friedrichsdorf, Germany), and medium-chain triglycerides (MCT OIL; SHS Nutricia/Danone®, GmbH, Friedrichsdorf, Germany) are shown in Table 1.

**Table 1:** Energy and nutrient contents of the human milk fortifier (Aptamil FMS®), modular protein hydrolysate (Aptamil Protein Supplement®) and modular medium-chain triglycerides (MCT OIL SHS®) used

Product	Energy (kcal)	Protein (g)	Fat (g)
Aptamil FMS® (*per* 100 g)	347	25.2	0
Aptamil Protein Supplement ® (*per* 100 g)	328.4	82.1	0
MCT OIL SHS® (*per* 100 mL)	855	0	95

For each infant, the study period was from full enteral feeding to 35 weeks plus 6 days PMA, or to the PMA at which the infant became breastfed and the OMM analysis suspended. For the whole sample, the mean (SD) study period was of 29 (8) days.

The measured composition of OMM was retrospectively compared with recent longitudinal data from a meta-analysis on preterm OMM composition [15] and measured composition of DHM was compared with reported composition of pooled DHM [12], using the t test. The actual energy and macronutrient intakes were calculated based on measured OMM and DHM compositions *plus* compositions provided by the manufacturers of the HM fortifier, modular protein and modular fat. As OMM composition measurements were not always possible in this study, mixed models were used for imputation of missing values. These models used logarithmic transformations of preterm OMM energy and macronutrient measured concentrations (as non-linear dependent variables), the postnatal days as the fixed effect and each case as a random effect. The composition of delivered DHM has always been measured. The assumed energy and macronutrient intakes were calculated based on the aforementioned reported compositions of preterm OMM [15] and pooled DHM [12] *plus* the compositions of HM fortifier, modular protein, and fat. The actual energy and macronutrient intakes were compared with the assumed intakes using the Wilcoxon matched-pairs signed ranks test.

Results are expressed as median (interquartile range - IQR) and significance was considered as *p* < 0.05. Statistical analysis was performed using SPSS (version 13, SPSS Inc., Chicago, IL).

## Results

One thousand one hundred eighty-one daily pools of HM have been individually delivered to infants, and the composition of 1021 (86.5%) of these pools (905 OMM and 116 DHM) has been analyzed. Concerning HM with analyzed composition, in 99.4% of study days, infants were exclusively OMM fed, in 4.3% OMM *plus* DHM fed, and in 0.3% exclusively DHM fed.

When compared with reported longitudinal OMM composition [15], measured protein content was always significantly lower, energy and carbohydrate contents significantly lower only from the second postnatal week, and fat content significantly lower only from the third postnatal week (Table 2).

**Table 2:** Comparison between measured composition of OMM and data from a meta-analysis [15]

Postnatal day	Measured energy (Kcal/100 mL)	Reporte denergy (Kcal/100 mL)	Measured protein (g/100 mL)	Reported protein (g/100 mL)	Measured fat (g/100 mL)	Reported fat (g/100 mL)	Measured carbohydrate (g/100 mL)	Reported carbohydrate (g/100 mL)
Day 1–3 Mean (SD)	*N* = 73 61.0 (14.00)	*N* = 143 58.80 (7.91)	N = 73 1.84** (0.53)	*N* = 163 2.57 (1.44)	N = 73 3.01* (1.15)	*N* = 173 2.52 (0.98)	N = 73 6.25 (1.52)	N = 143 6.2 (0.92)
Day 4–7 Mean (SD)	*N* = 96 64.5 (10.49)	N = 96 67.9 (14.1)	N = 96 1.89* (0.49)	*N* = 44 2.11 (0.44)	N = 96 3.34 (1.11)	*N* = 110 3.31 (1.27)	N = 96 6.34 (1.54)	*N* = 87 6.17 (0.49)
Week 2 Mean (SD)	*N* = 157 62.0** (11.75)	*N* = 417 69.1 (10.1)	N = 157 1.70** (0.50)	*N* = 383 1.98 (0.68)	N = 157 3.19 (1.00)	*N* = 426 3.19 (1.04)	N = 157 6.32** (1.93)	*N* = 389 6.72 (0.46)
Week 3–4 Mean (SD)	*N* = 203 63.9** (9.62)	*N* = 481 70.87 (9.34)	N = 203 1.61 (0.46)	*N* = 528 1.6 (0.5)	N = 203 3.32** (0.87)	*N* = 485 3.83 (1.01)	N = 203 6.53** (1.26)	*N* = 464 7.05 (0.51)
Week 5–6 Mean (SD)	*N* = 84 67.6** (7.92)	*N* = 354 73.97 (9.1)	N = 84 1.55* (0.60)	*N* = 330 1.43 (0.25)	N = 84 3.63* (1.00)	*N* = 371 4.04 (0.91)	N = 84 6.71** (1.42)	N = 354 7.14 (0.36)
Week 7–9 Mean (SD)	N = 42 64.4** (9.81)	*N* = 239 74.24 (8.77)	N = 42 1.18** (0.23)	*N* = 223 1.34 (0.2)	N = 42 3.63* (0.85)	*N* = 236 4.21 (0.92)	N = 42 6.40** (1.16)	*N* = 235 7.13 (0.38)

t test; * *p* < 0.001; ** *p* < 0.0001 Abbreviations: *OMM* own mother’s milk

Compared with reported pooled DHM composition [12], measured energy and fat contents were significantly lower and carbohydrate significantly higher, without differences in protein content (Table 3).

**Table 3:** Comparison between measured composition of DHM and reported pooled DHM [12]

	Measured Samples = 116		Reported Samples = 179		*p* ‡
	Mean	SD	Mean	SD	
Energy (kcal/dL)	60.0	3.6	66.0	12.0	< 0.0001
Protein (g/dL)	0.9	0.2	0.9	0.4	NS
Fat (g/dL)	2.8	0.3	4.0	1.4	< 0.0001
Carbohydrates	7.1	0.4	6.6	0.7	< 0.0001

‡ t test Abbreviations: *DHM* donated human milk

**Fig. 1:**
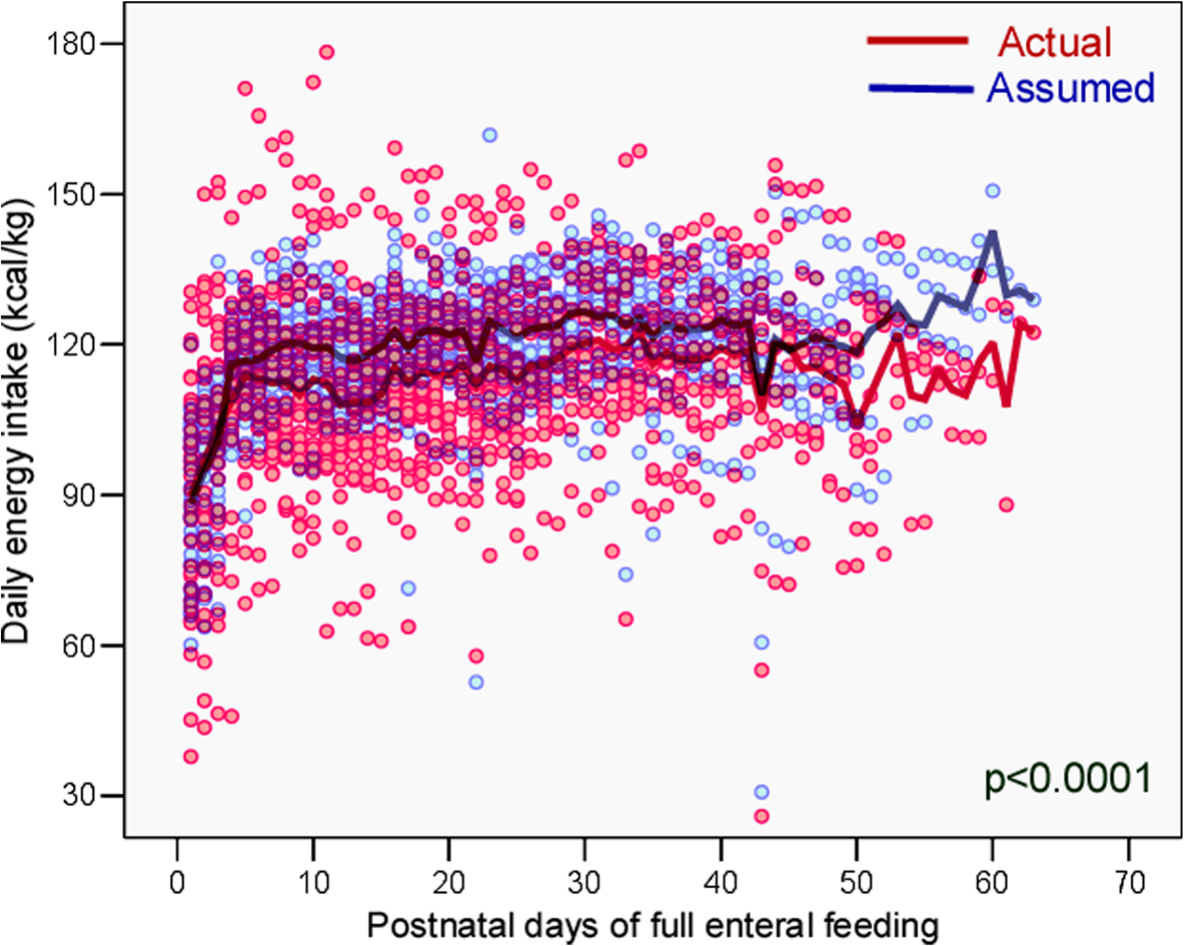
The actual daily total energy intake provided by exclusive enteral feeding was significantly lower than the assumed energy intake, during the study period

**Fig. 2:**
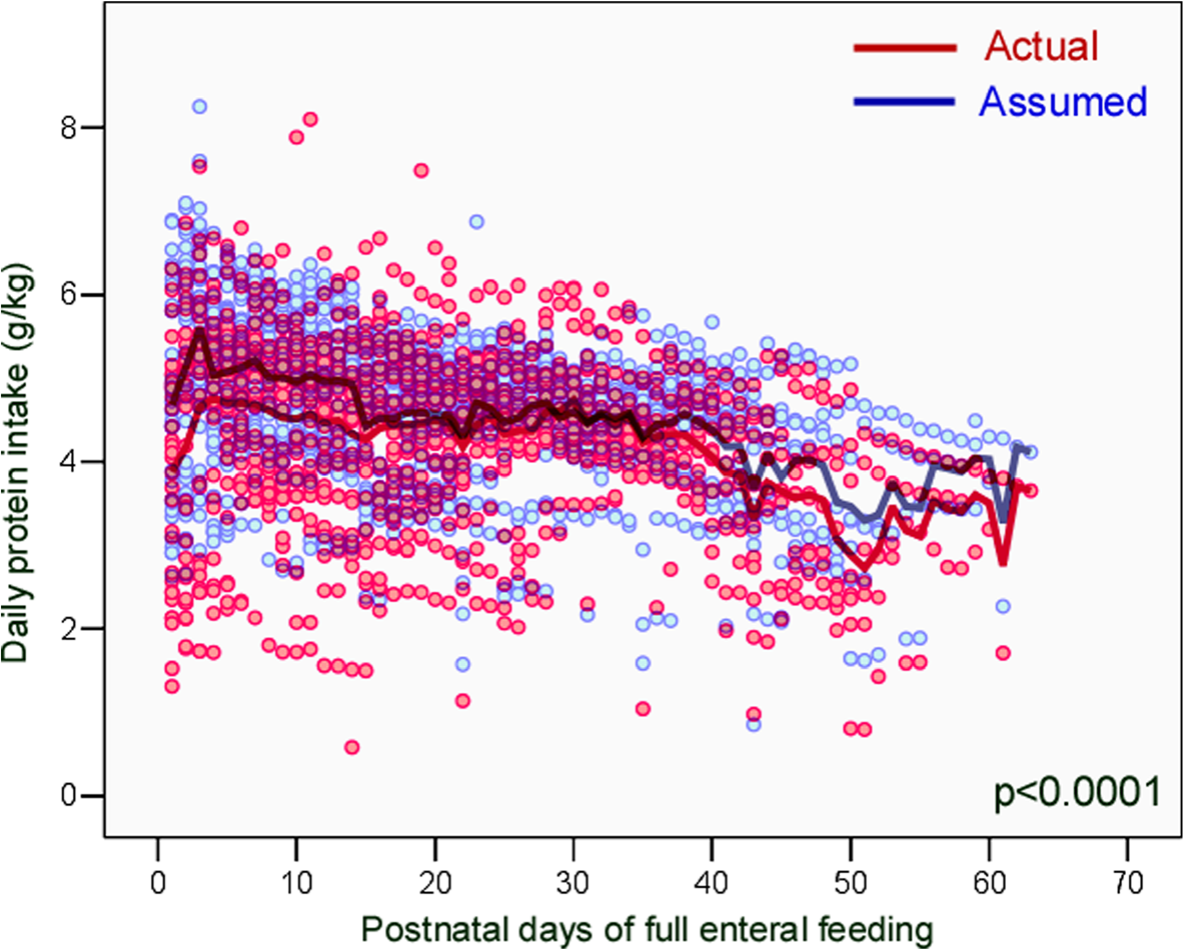
The actual daily protein intake provided by exclusive enteral feeding was significantly lower than the assumed protein intake, during the study period

**Fig. 3:**
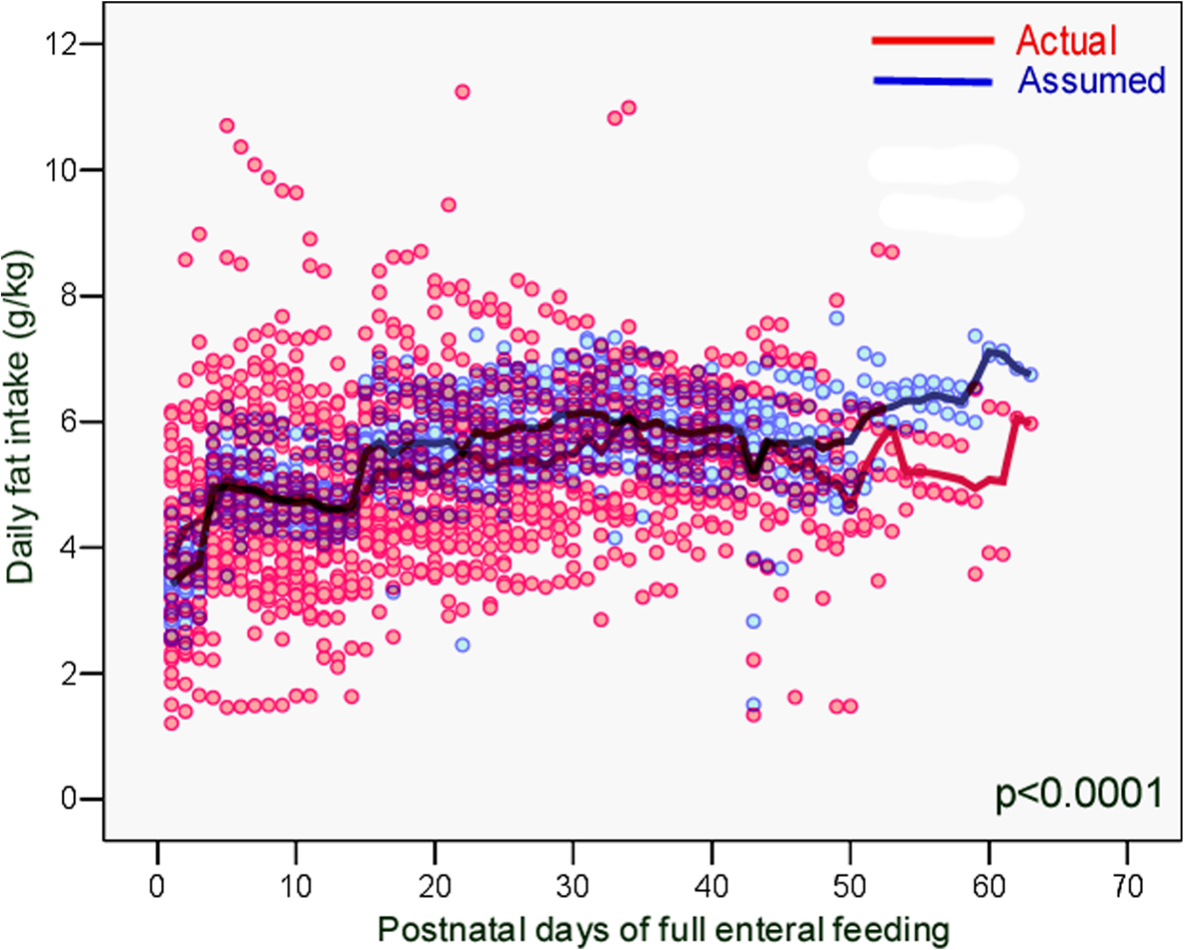
The actual daily fat intake provided by exclusive enteral feeding was significantly lower than the assumed fat intake, during the study period

**Fig. 4:**
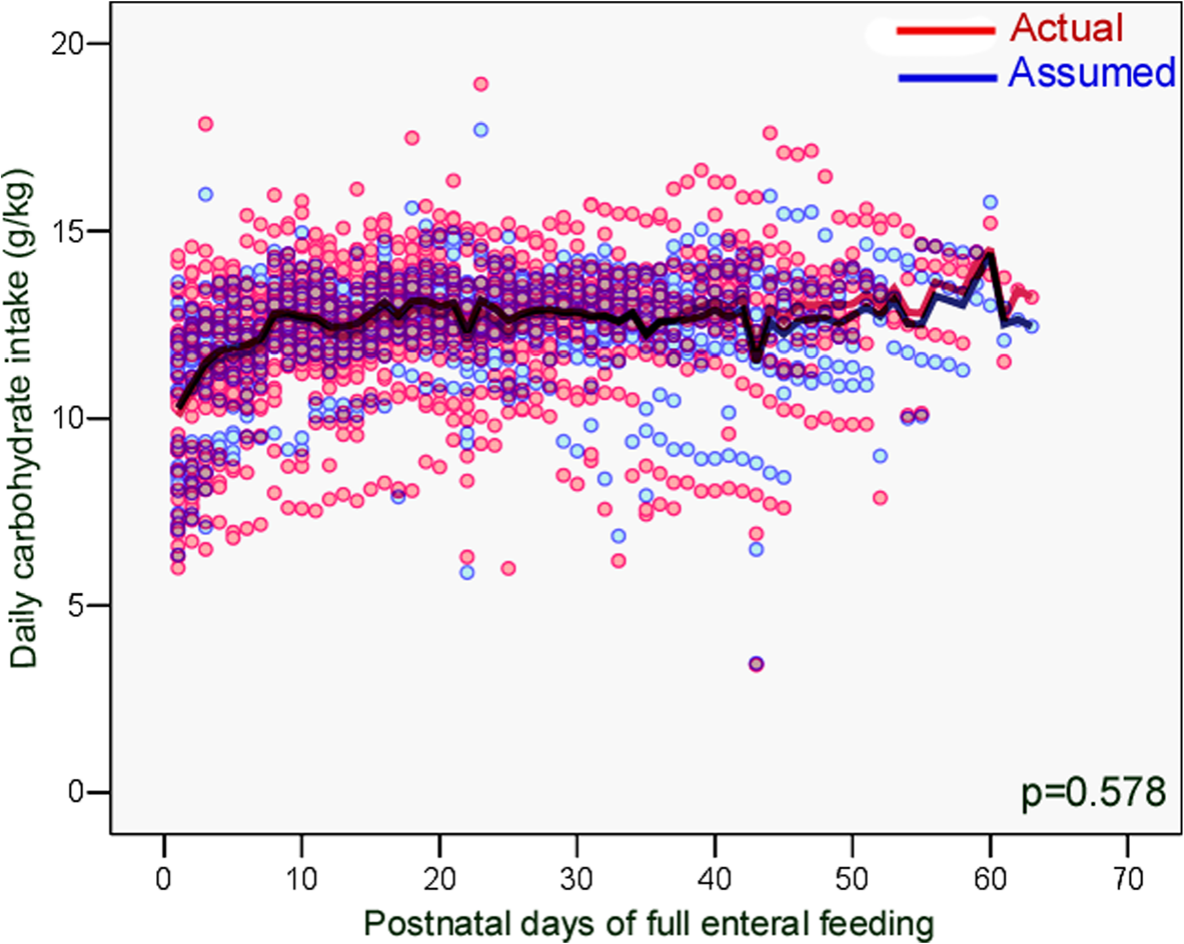
The actual daily carbohydrate intake provided by exclusive enteral feeding did not differ from the assumed carbohydrate intake, during the study period

The actual energy, protein, and fat intakes were significantly lower than the assumed intakes (Figs. 1, 2 and 3), without significant differences in carbohydrate intake (Fig. 4); the actual PER was significantly higher than the assumed value (Table 4).

**Table 4:** Comparison between actual and assumed daily intakes during the study period

Nutrients	Actual daily intake median (IQR)	Assumed daily intake median (IQR)	*p* ‡
Energy (kcal/kg)	113.3 (102.8–123.7)	120.7 (113.6–127.3)	< 0.0001
Protein (g/kg)	4.45 (3.70–5.07)	4.73 (4.09–5.26)	< 0.0001
PER (protein g/100 kcal)	4.2 (3.6–4.7)	4.0 (3.5–4.6)	0.011
Fat (g/kg)	4.96 (4.19–6.00)	5.35 (4.76–5.93)	< 0.0001
Carbohydrates (g/kg)	12.64 (11.63–13.55)	12.71 (12.05–13.29)	0.578

Abbreviations *IQR* interquartile range, *PER* protein-to-energy ratio ‡ Wilcoxon signed rank test

## Discussion

In this study, energy and macronutrient intakes were mainly influenced by OMM composition, since infants received DHM in a very small percentage of the days. Compared with recently reported values [15], measured OMM protein content was significantly lower, and energy and other macronutrients became significantly lower from the second or third postnatal week. When fortifying the HM, we targeted the minimum recommended intakes, concerned to not exceed the recommended threshold of 400 mOsm/L for infant feeds, which could easily occur with the addition of both fortifier and modular protein to HM [20]. The fortification method was guided by our unit protocol that inappropriately assumed lower energy and protein contents than reported in literature. In consequence, the HM fortification did not compensate the low measured OMM energy and macronutrient contents, and our fortification method resulted in actual energy, protein, and fat intakes significantly lower than the assumed values. Particularly, the actual energy intake was relatively lower than the low actual protein intake, reflected by a higher actual PER compared with the assumed PER.

In a previous study on the same infants [6], it was found that minimum recommended intakes were achieved only in 63.6% of infants for protein, 15.2% for energy, and 93.9% for PER; this was associated with a low weight gain velocity (mean 10.1 g/kg/day) compared with that described in similar populations [21].

In surveys, enteral nutritional practices used for preterm infants were found to be quite heterogeneous [22, 23]. Moreover, varied methods of HM fortification have been used [10]. This may explain different results on energy and nutrient intakes [8, 13] and growth [8, 11, 14] reported in studies that have compared fortification methods based on assumed HM composition with those relying on measured HM composition.

de Halleux et al. (2013) compared energy and nutrient intakes between individualized fortification relying on measured HM composition and standard fortification based on assumed HM composition [13]. The individualized fortification resulted in actual energy and fat intakes higher than the assumed values and actual protein intake and PER lower than the assumed values. This seems contrary to our results and may be explained by different fortification methods used. In standard fortification, de Halleux et al. [13] added to HM a fixed recommended amount of fortifier; the individualized fortification was performed after analysis of HM and its fat content was first adjusted up to 4.0 g/100 mL using modular fat; subsequently, HM fortifier was added to achieve a protein intake of 4.3 g/kg/d. This method resulted in actual mean intakes of 140 Kcal/kg/d of energy and 4.25 g/kg/d of protein. In our study, modular protein and fat supplements were added to standard fortified HM to achieve intakes of 4.0 g/kg/d of protein and 110 Kcal/kg/d of energy. Compared with de Halleux et al. data [13], our nutritional strategy resulted in a lower actual mean energy intake (113 Kcal/kg/d), but slightly higher actual mean protein intake (4.45 g/kg/d). This might be explained by excessive protein added to reach the minimum targeted intake according to our unit protocol that inappropriately assumed a lower protein concentration than described for preterm OMM [15].

McLeod et al. (2016) [8], in a trial including 40 very preterm infants, compared routine fortification based on assumed HM composition with targeted fortification relying on measured HM composition. Human milk fortifier, modular protein and carbohydrate plus fat supplement were used for HM fortification. In HM, mean measured protein content (1.6 g/100 mL) was higher than the assumed value (1.4 g/100 mL), which is contrary to our result. Despite differences in measured and assumed protein content, neither significant differences in energy and macronutrient intakes, nor in weight gain velocity were found between groups. Compared with our study, McLeod et al. (2016) targeted the intakes to upper daily values of protein (3.8–4.4 g/kg) and energy (130–150 Kcal/kg) [8].

Limitations of our study should be acknowledged. First, for 19.4% of the study days, OMM with unknown composition was delivered to infants, potentially affecting the calculation of nutrient intakes. To mitigate this inconvenience, mixed models for imputation of missing nutrient values of OMM were used. In a previous study, we found good agreement between curves obtained with model-predicted data and those from a meta-analysis on preterm OMM composition [6]. Second, processes of freezing, thawing, and homogenization may have caused a reduction in fat and protein concentrations [24]. Nevertheless, processing of HM in our study was also used in studies whose results we compared [8, 12, 13].

## Conclusion

In this study we compared assumed with actual macronutrient intakes in a cohort of very preterm infants fed HM, predominantly fed OMM. When fortifying the HM, we chose conservative targeted nutrient intakes hoping not to exceed the osmolarity recommended for infant feeds. Actual energy, protein and fat intakes in OMM were significantly lower than assumed. This resulted in inadequate intake using our fortification method, that did not compensate the suboptimal measured energy and macronutrient contents of OMM delivered.

Further studies comparing assumed with target fortification are needed to determine safe upper limits of assumed fortification to guide clinicians in their fortification practice [8]. Meanwhile, the target fortification tailored to the infant’s needs is the reference to achieve the recommended nutrient intakes, although this method is time consuming, laborious and HM analyzers are commonly unavailable.

## Abbreviations

DHM: Donated human milk
HM: Human milk
NICE: National Institute for Health and Care Excellence
OMM: Own mother’s milk
PER: Protein to energy ratio
PMA: Postmenstrual age
